# Long-Term Lateral Semicircular Canal Function in Children with Cochlear Implants: Results of Video Head Impulse Test

**DOI:** 10.3390/ejihpe11010002

**Published:** 2021-01-09

**Authors:** Nader Nassif, Cristiano Balzanelli, Luca Oscar Redaelli de Zinis

**Affiliations:** 1 Department of Pediatric Otorhinolaryngology, ASST Spedali Civili, 25123 Brescia, Italy; nadernassif@alice.it; 2 Vertigo Center-San Bernardino Policlinic of Salò, 25087 Brescia, Italy; balzanelli.cristiano@gmail.com

**Keywords:** cochlear implant, video head impulse test, children

## Abstract

In children with profound deafness, bilateral cochlear implant (CI) is an effective, established procedure. However, its safety on vestibular function has recently been debated. The goal of this study is to evaluate the long-term lateral semicircular canal high-frequency vestibulo-oculomotor reflex (LSC HF VOR) in children with CI by video head impulse testing (vHIT). This is a cross-sectional study assessing a cohort of children who received either a unilateral (12) or a bilateral (12) cochlear implant (CI), compared with a control group of 12 normal-hearing children. No significant LSC HF VOR gain difference was found between CI users and controls. In the unilaterally implanted group, the LSC HF VOR gain measured in the “CI-ON” condition was significantly higher than in the “CI-OFF” condition, both in the implanted and in the non-implanted ear. In the bilaterally implanted group, the difference between the two conditions was not significant. Our results do not show any impairment of LSC HF VOR function in children with CI compared to normal-hearing children in the long-term period. This suggests that both unilateral and simultaneous/sequential bilateral CI are procedures that do not impair HF LSC long-term function when analyzed by vHIT.

## 1. Introduction

Bilateral cochlear implant (CI) has become the recommended indication for childhood deafness thanks to the growing attention to the importance of binaural hearing. Consequently, the safety of bilateral cochlear implantation has become an important issue and the impact of cochlear implantation on vestibular function is now a matter of discussion.

Some papers analyzing the impact of unilateral cochlear implantation in children report abnormal results from vestibular assessment in 50% to 68% of cases [1,2,3]. It could be debatable if these abnormal results are clinically relevant or, on the other hand, if the clinical effect could be underestimated. Nonetheless, the long-term function of vestibular labyrinth after cochlear implantation in children has not been extensively investigated in the literature.

Video head impulse testing (vHIT) is a method for quantitative measurement of eye movements during head thrust testing and has been gaining popularity in the last years. It is effective for assessing the semicircular canal function in children in a fast, easy, and precise mode, with potential advantages over the rotary chair and caloric testing [4,5,6].

In a previous paper, we reported that in implanted children a significant impairment of lateral semicircular canal (LSC) function compared to normal hearing children was not observed [7]. In this new study, we analyzed a greater number of bilateral implanted children to evaluate if long-term LSC function was still preserved and if there was a difference according to type of approach and electrode used.

## 2. Materials and Methods

### 2.1. Patients

This is a cross-sectional study assessing a cohort of children who received either a unilateral (group A) or a simultaneous/sequential (group B) CI, at least 4 years before the moment of the analysis. A group of age- and sex-matched healthy children with normal hearing (NH) and normal vestibular function were included as controls. Surgery was performed using the same technique, consisting of mastoidectomy and posterior tympanotomy, whereas access to the basal cochlear turn was achieved either via a round window or via promontorial cochleostomy. Three different brands of implants and electrodes were used. Syndromic children, children with cochlear malformation or a history of infectious disease such as meningitis or Cytomegalovirus, children who received revision surgeries, and children with peri- or post-operative complications were not included in the study. The patients’ medical history was collected from charts looking for vestibular signs and symptoms before and after the operation.

### 2.2. Vestibular Evaluation

Clinical vestibular function testing of the study was performed by one of the authors (CB), who is an expert in vestibular disorders in children. Both implanted and NH children received a vestibular function test battery, including the following.

#### 2.2.1. Basic Clinical Examination

- Vestibular examination by means of an infrared goggles system: spontaneous nystagmus; positional nystagmus (left and right side, supine); positioning nystagmus (Dix-Hallpike maneuver, roll-supine test); head-horizontal shaking nystagmus;
- Balance evaluation: stretched arms test; Romberg test; Unterberger Fukuda test;

#### 2.2.2. Video Head Impulse Test

- High-frequency stimulation (HF) of lateral semicircular canal (LSC) Vestibulo-Ocular Reflex (VOR) gain assessment by means of a vHIT, which represented the main outcome measure because of its feasibility in implanted children and high sensitivity in detecting canal function [5,6]. In implanted subjects, vHIT was performed on both sides and in the “CI-ON” and “CI-OFF” conditions.

The infrared goggles system utilized was the Ulmer VNS 3x HF, Synapsis, France [general technological characteristics: horizontal field of vision 100°, vertical 55°; material: Polyoxymethylene and Polyvinyl chloride(foams); weight (without cable): 150 gr; camera: power supply 8.6 V DC, sensor type complementary metal oxide semiconductor, resolution pixels, sensibility 0.2 lux, adjustable zooming yes, power consumption 50 ma; infra-red light: wavelength 950 nm, power 2.55 mw per LED, lighting cone 40° per LED; transmitter: transmitter frequency 2414.5 MHz and/or 2470.5 MHz, receiver: receiver frequency 2414.5 MHz and/or 2470.5 MHz, power supply 9 V (500 mA)].

The vHIT was administered using ICS Impulse, GN Otometrics A/S, Denmark [technological characteristics: inputs head: 9 axis motion sensor; inputs eye: monocular (right eye only); sampling rate: 250 fps-impulse; eye tracking: 100 × 100 pixels-impulse; OTOsuite^®^ vestibular software: Windows graphical user interface; High-Performance Analysis software; database; storage of test data; sophisticated patient and test data management; vision denied for testing in complete darkness; patient calibration: goggles have 2 built-in calibration lasers]. Subjects were seated 1 m from a visual target mounted at eye level on the wall. The examiner stood behind the participant and delivered randomized (timing and direction) head impulses (120°/sec up to 250°/sec peak head velocity) in the plane of the lateral semicircular canal, until approximately 20 acceptable head impulses were recorded. Responses were considered in terms of gain (eye velocity/head velocity), which was automatically calculated by the device software by dividing the area under the curve for eye velocity (with reset saccades removed) by the area under the curve for head velocity [4].

### 2.3. Statistical Analysis

Statistical analysis was performed using the SPSS statistical package (SPSS Inc., Chicago, IL, USA) to perform: (1) chi-square test to compare categorical variables; (2) Mann–Whitney U test to compare two unrelated populations on one continuous variable; (3) Kruskal–Wallis test to compare three unrelated populations on one continuous variable; and (4) Wilcoxon test to compare two related populations on one continuous variable. The results were expressed as medians and interquartile ranges (IQR). 

## 3. Results

### 3.1. Groups of Patients

A total of 36 subjects were included in the study. There were 7 females and 5 males in group A (unilateral CI), 6 females and 6 males in group B (simultaneous/sequential CI), and 5 females and 7 males in the control group (*p* = 0.7). The mean age at first cochlear implantation was 6 years (range 1-13 years) and the mean age at evaluation was 10 years (range 5–17 years). Median (IQR) age was 10.5 (7.5–14) years in group A, 8 (6–10) years in group B, and 9.5 (7.5–10) years in controls (*p* = 0.9).

### 3.2. Etiology

The etiology and onset of SNHL were congenital connexin CX26 positive in 10 patients and idiopathic in 14 cases. All children underwent both preoperative CT scan and MRI with normal radiological findings.

### 3.3. Surgical Approach

The round window approach was used in 22 ears (61%) and promontorial approach (anterior-inferior cochleostomy to the round window) in 14 (39%). A perimodiolar electrode was inserted in 27 ears (75%) and a straight electrode in 9 (25%). All had complete insertion of the electrode.

Results of clinical vestibular and balance function tests were normal in both the CI group (both CI-ON and CI-OFF conditions and in the non-implanted ear) and in NH subjects. 

Furthermore, vertigo and balance problems were never reported in medical charts or referred from parents of the implanted children both pre- or post-operatively. The vHIT procedures were well tolerated by all children. 

Median (IQR) gain (vHIT HF LSC VOR) of controls was 0.90 (0.86–0.94): the difference between right ears [0.90 (0.87–0.95)] and left ears [0.90 (0.86–0.93)] was not significant (*p* = 0.8). Gain distribution difference between implanted ears and ears of non-implanted subjects was never significant when the implants were ON or when the implants were OFF (Table 1). There was a significant increase of contralateral ear gain when the implant was ON compared to the gain of control ears (Table 1). Median gain of all implanted ears was significantly different with implant ON and OFF (Table 1). Median gain of group A was significantly increased with implant ON vs. OFF both for implanted and non-implanted ears, while the difference of gain distribution between implanted and non-implanted ear was never significant (Table 1). Median gain of implanted ears in group B was increased without reaching significance with implant ON vs. OFF (Table 1). 

Median (IQR) gain did not significantly differ according to the type of surgical approach to the cochlear basal turn and type of electrode (Table 2).

## 4. Discussion

Hearing impairment has been associated with vestibular impairment and motor performance disturbance in children [8,9]. When hearing-impaired children who are candidates for cochlear implantation have been studied for vestibular function, it has been shown that abnormal otolith responses and LSC dysfunction including areflexia with caloric testing vary between 50% to 68% [1,3,10], with no correlation with vestibular malformations or hearing loss etiology [1].

The mechanism of CI-determined vestibular deficits is not yet completely known. The literature offers several explanations: a direct trauma provoked by the surgical maneuvers during electrode insertion [11]; an inflammatory process that may be a result of the presence of a foreign body causing a fibrosis reaction and, therefore, labyrinthitis [12]; the opening of the cochlea that alters the homeostasis of the cochlea with perilymphatic loss or endolymphatic hydrops causing inner ear liquid imbalance [13,14]; the electrical vestibular stimulation by the implant that may impair vestibular function [15].

When vestibular function has been objectively tested in children, a performance decrease after cochlear implantation, in most cases unilateral, was observed up to 100% of implanted ears by several authors [1,2,3,4,16,17,18,19].

Dynamic balance function test showed poorer performance by children with unilateral CI compared to their peers with normal hearing [16]. Canal VOR modification was observed in 39% and vestibular evoked myogenic potentials (VEMP) responses in 55% after unilateral implant in children with 10% of vestibular loss [1]. Reduced vestibular function on the CI side, with 52% abnormal VOR results, 80% abnormal VEMP findings, and 39% abnormal computerized dynamic posturography results, was observed [2]. Postural control on posturography was poorer in adolescents with unilateral CI [17]. VEMP responses on the implanted side were lost in all children analyzed by Psillas et al. [18]. Various anomalies of the vestibular function were observed after unilateral cochlear implant in 50% of children [3]. The oVEMPs and cVEMPs on the operated side disappeared in 82% of children [19]. When VEMPs could be elicited on the operated side, the parameters of waveforms showed abnormal changes, including threshold elevation and amplitude decrease [19]. Finally, Janky and Givens [4] were the first to use vHIT to test semicircular canals function after uni- or bilateral CI in children. Validating their results by comparing the rotary chair test, they observed 55% of abnormalities of lateral semicircular canal [4]. They also observed similar values for VEMP testing [4].

Despite the high probability of vestibular testing abnormalities after CI in children, vestibular anomalies seem to be clinically silent: in fact, they do not correlate with vestibular symptoms and do not interfere with daily activities. Moreover, they can be detected only by systematic testing [1,2,17,18]. This paucity of clinical signs has been explained by the rapid compensation of sensory deficits in children [1,2,17,18]. In fact, only transient vestibular complaints after cochlear implantation have been reported, varying from 1% to 23% in children [16,20,21].

As is well known, evaluation of vestibular function in children is a challenging task, especially in those below 5 years of age [1,3,5], and the compliance of the child usually depends on the test administered. Some authors have found that Electronystagmography and caloric testing are not well tolerated in children, whereas VEMPs are well tolerated even if they can be accomplished in about 30 min [2]. vHIT provides canal-specific testing for both unilateral and bilateral peripheral vestibular losses [5]. vHIT does not induce vertigo or nausea and is done in an open, lighted room, which makes it much more ‘‘child-friendly’’ [5]. Moreover, the analysis of HF LSC VOR studies the canal that is used in about 80% of head movements, with frequency of stimuli of everyday life, whereas caloric testing involves lower, non-physiological frequencies.

We selected a homogenous group of patients who underwent CI in our department for non-syndromic sensorineural hearing loss at least 4 years before; they were evaluated by vHIT to observe the LSC function. The compliance of children to the test was very good. In no case was the vHIT interrupted because it does not induce vertigo or nausea and takes only a few minutes to be completed. Furthermore, it was performed by an expert vestibologist (CB), offering a possibility to obtain results without repeating the maneuvers several times and avoiding artifacts in the recorded traces.

This study investigated unilaterally and bilaterally implanted children, comparing the “CI-ON” and “CI-OFF” configurations, i.e., performing the test with the CI switched on and switched off, respectively. Our results show that LSC HF VOR gain distribution difference between implanted ears and ears of non-implanted subjects both when the implants were ON or OFF was never significant, except in case of implant ON when there was a significant increase of contralateral ear gain. There was no significant difference in LSC HF VOR gain between the implanted and the non-implanted side in unilaterally implanted children. Every condition with implant ON compared to implant OFF resulted in increased LSC HF VOR gain. Finally, gain distribution difference according to type of surgical approach to the cochlear basal turn and type of electrode was not significant. These results in a larger group of patients confirmed the observation of our previous study [7].

The interpretation of gain increases while the implant was in the “ON” modality is still debated in the literature. Increased LSC HF VOR gain when the implants were ON suggests a direct effect of the electrical stimulation on vestibular receptors and vestibular nerve afferent neurons. This is consistent with recent studies on vestibular implants, suggesting that LSC HF VOR gain can be increased by electrodes stimulating the semicircular canals in adult patients with bilateral vestibular loss [22,23]. It is possible that the spread of excitation of electrodes in the basal turn of the cochlea, which is the nearest to the vestibule, may reach the vestibular labyrinth, thus eliciting an increase of LSC HF VOR gain, although the physiologic mechanism is still unknown. Contralateral LSC HF VOR gain modification explanation still remains controversial [1,10,24,25,26]. From a neurophysiological point of view, it is known that the vestibular type I cells of the ampullae present their own resting-rate frequency discharge (about 90 spikes/sec in monkeys, but it is uncertain in humans), and that vestibular stimulation works in a “push-pull” mode, so that ciliated cells of both sides are activated during every single-side stimulation depends on the frequency–amplitude–direction of head rotation [27,28,29]. In our study, we can assume that CI activation provokes a bilateral increase in the resting rate of ciliated cells thanks to various mechanisms, such as the push-pull mode of the vestibular HF stimulation and vestibular nuclei modulation. The final action of CI activation is a sensible and recordable ipsi- and contralateral increase of HF-VOR gain in the lateral semicircular canal. We are convinced that there are possibly other complex neuroplasticity mechanisms involved in the vestibular system during CI activation and further studies on greater numbers of patients are needed.

The most important limitation of our study is that the vHIT analysis of LSC function has not been compared with caloric testing since there is evidence that LSC HF VOR gain results abnormal only when vestibular deficit on caloric testing of the canal is higher than 40% [30]. It is likely that vestibular function could be better assessed with a test battery [31]. The decision to perform vHIT without comparison with caloric testing was based on validation of vHIT testing in children made by other investigators who also pointed out the unpleasant sensations often caused by caloric and rotational testing in children [4,5,6]. A second limitation is the cross-sectional nature of the analysis so that we cannot compare the pre-operative, postoperative, and long-term post-operative vestibular function in our CI group.

## 5. Conclusions

The potential balance impairment in children with sensorineural hearing loss undergoing uni- or bilateral CI is still under debate. The high plasticity of the central nervous system in children produces the prompt activation of functional and sensory substitution recovery strategies that prevent impairment of ordinary movements of daily life, such as walking, running, and playing. Our results on implanted children show no significant impairment of LSC HF VOR function compared to NH subjects in the long-term. This suggests that both unilateral and simultaneous bilateral CI are procedures that do not impair long-term HF LSC function when analyzed by vHIT. Further studies with longitudinal complete vestibular testing (calorics, saccular-utricular testing, and vertical canals vHIT) are required to substantiate our results.

## Figures and Tables

**Table 1:** Median (IQR) vestibulo-ocular reflex (VOR) gain of controls and implanted patients.

			Median (IQR)	*p* (ON vs. OFF)	*p* (IE vs. NIE)	*p* (IP vs. C)
Controls (US)	All ears (24)		0.90 (0.86–0.94)			
		IC ON	0.95 (0.87–1.06)			0.1
Group A + B	IE (36)			0.001		
		IC OFF	0.89 (0.82–0.97)			0.4
		IC ON	1.01 (0.89–1.11)		0.4	0.07
Group A	IE (12)			0.02		
		IC OFF	0.89 (0.82–0.96)		0.3	0.4
		IC ON	0.98 (0.88–1.14)			0.049
Group A	NIE (12)			0.03		
		IC OFF	0.91 (0.86–0.99)			0.07
		IC ON	0.93 (0.87–1.03)			0.3
Group B	All ears (24)			0.08		
		IC OFF	0.90 (0.87–0.99)			0.9

IE: implanted ears; NIE: non-implanted ears in unilateral CI; C: controls; IP: implanted patients; US: unimplanted subjects.

**Table 2:** Median (IQR) VOR gain according to the type of approach and electrode.

	CI ON	CI OFF
Round window approach (22)	0.96 (0.88–1.07)	0.86 (0.82–0.96)
Promontorial approach (14)	0.93 (0.87–1.00)	0.93 (0.83–0.98)
*p*	0.4	0.3
Perimodiolar electrode (27)	0.93 (0.88–1.05)	0.92 (0.83–0.98)
Straight electrode (9)	0.97 (0.81–1.06)	0.83 (0.78–0.87)
*p*	0.9	0.8

## Data Availability

The data presented in this study are available on request from the corresponding author. The data are not publicly available due to underage subjects.
